# Modelling reactive case detection strategies for interrupting transmission of *Plasmodium falciparum* malaria

**DOI:** 10.1186/s12936-019-2893-9

**Published:** 2019-07-30

**Authors:** Theresa Reiker, Nakul Chitnis, Thomas Smith

**Affiliations:** 1 Department of Epidemiology and Public Health, Swiss Tropical and Public Health Institute, 4051 Basel, Switzerland; 2 University of Basel, Petersplatz 1, Basel, Switzerland

**Keywords:** Simulation, Targeting, Elimination, Surveillance, Response

## Abstract

**Background:**

As areas move closer to malaria elimination, a combination of limited resources and increasing heterogeneity in case distribution and transmission favour a shift to targeted reactive interventions. Reactive case detection (RCD), the following up of additional individuals surrounding an index case, has the potential to target transmission pockets and identify asymptomatic cases in them. Current RCD implementation strategies vary, and it is unclear which are most effective in achieving elimination.

**Methods:**

OpenMalaria, an established individual-based stochastic model, was used to simulate RCD in a Zambia-like setting. The capacity to follow up index cases, the search radius, the initial transmission and the case management coverage were varied. Suitable settings were identified and probabilities of elimination and time to elimination estimated. The value of routinely collected prevalence and incidence data for predicting the success of RCD was assessed.

**Results:**

The results indicate that RCD with the aim of transmission interruption is only appropriate in settings where initial transmission is very low (annual entomological inoculation rate (EIR) 1–2 or prevalence approx. < 7–19% depending on case management levels). Every index case needs to be followed up, up to a maximum case-incidence threshold which defines the suitability threshold of settings for elimination using RCD. Increasing the search radius around index cases is always beneficial.

**Conclusions:**

RCD is highly resource intensive, requiring testing and treating of 400–500 people every week for 5–10 years for a reasonable chance of elimination in a Zambia-like setting.

**Electronic supplementary material:**

The online version of this article (10.1186/s12936-019-2893-9) contains supplementary material, which is available to authorized users.

## Background

One of the great challenges of malaria control in the face of decreasing transmission is onwards-transmission by asymptomatic infections [1], which are not detected by traditional interventions. Furthermore, heterogeneity in incidence and transmission and spatial and temporal clustering of cases [2] make mass interventions cost-inefficient, increasingly ineffective and unsustainable in low-transmission settings. The World Health Organization (WHO) elimination guide [3, 4] addresses this need to adapt intervention strategies in the elimination phase to highly targeted, locally adapted measures to track down foci of transmission. Surveillance-based strategies, such as (re)active case detection (RCD) serve as key tools in following these aims and optimizing resource allocation.

In reactive strategies, when an index case of clinical malaria presents to a health facility, this triggers follow-up activities around the index case. Assuming that cases are geographically clustered and that presence or absence of symptoms is independent of surrounding cases, reactive interventions may thus allow targeted detection or intervention on symptomatic cases who do not seek care as well as asymptomatic cases. Although reactive strategies have high approval rates and have been implemented in a range of settings, in practice, many variations of strategies are employed globally. In low-prevalence urban areas of India, contacts of cases are screened [5]. In Southern Province, Zambia, all individuals within a 140 m radius of the index case are tested [6]. RCD has also been implemented in 13 of 14 countries in the Asia Pacific region including China [7]. China successfully adapted the so-called “1-3-7” strategy where malaria cases are reported within 1 day, their confirmation and investigation occurs within 3 days and the appropriate follow-up intervention to prevent onwards transmission occurs within 7 days. Follow-up interventions may include indoor residual spraying (IRS) or RCD within the household [7, 8]. Little information on the detailed implementation strategies is available for other programmes in the Asia Pacific region [9]. Overall, the range of possible surveillance-response combination strategies is too vast for systematically assessing different strategies across a wide range of settings. A comparison of RCD efficacy between the few existing field studies is difficult due to differences in contextual determinants (such as health system infrastructure, geography, demographic structure or transmission intensity), which may influence optimal strategies. In addition to this, none of the existing field studies have assessed the effect of RCD on transmission [10] and the sites in question are yet to reach elimination, making them unsuitable for deriving even case study estimates of the potential of different RCD strategies in achieving elimination. Even with a simple test-and-treat follow-up, the relative relevance of parameters, such as the numbers of index cases and follow-up individuals, and optimum strategies thus remain to be determined.

Previous work using a deterministic susceptible-infected-susceptible (SIS) model highlights the importance of prevalence at the beginning of the intervention and suggests that relative values of the number of index cases followed, ${\iota}$, and the number of neighbours in the search radius, $\nu$, affect equilibrium prevalence levels even when the total number of individuals screened (the product of $\iota$ and $\nu$) is the same. The proportion of all infections found per unit time appears to be the main determinant of reduction in prevalence [11]. These models highlight the main features of the dynamics of the system but cannot provide quantitative predictions applicable to specific settings as they do not incorporate stochastic events, the effects of seasonality, within host parasite dynamics, host immunity, dynamic numbers of index cases or dynamic testing rates. In this study, test-and-treat-based reactive case detection was implemented in OpenMalaria, a powerful, individual-based stochastic model that includes the above factors. OpenMalaria was parameterized using a data set from Southern Zambia, to provide a realistic setting for which the applicability of simpler models was evaluated. The relevance of different parameters in determining the proportion of runs where transmission is interrupted was assessed by carrying out simulations utilizing different RCD strategies across entomological and health care settings.

Two large simulation experiments are reported in this paper. The first experiment is used to characterize settings where RCD alone can lead to interruption of transmission, assess which strategies are most efficacious in the different settings, and determine the sensitivity of success to programme specific parameters (follow-up capacity vs. search radius). The second experiment is used to assess the time to interruption of transmission and determine whether success is predictable through routinely collected data. Because the simulations are stochastic, the results were analysed using conventional statistical models and machine learning techniques, treating them as a large real-world experiment.

## Methods

### Transmission and disease model

The impact of RCD was simulated using OpenMalaria (https://github.com/SwissTPH/openmalaria.wiki.git), a modelling platform allowing for individual-based stochastic models of malaria dynamics in humans [12], linked to a periodically-forced deterministic model of malaria in mosquitoes [13]. Further details about the model have been previously published [14]. In brief, OpenMalaria captures different clinical presentations of malaria in individual humans as well as vector ecology across a range of species and *Plasmodium falciparum* dynamics in both humans and mosquitoes, allowing for simulations of interventions in comparatively realistic settings. Since blood stage parasite densities are tracked, the model allows for case management based on simulated events dependent on individual patient parasite densities. The simulated human population is updated every 5 days with multiple outcomes, including clinical incidence, the total number of infections, and the infectiousness to mosquitoes, which depends on the recent history of parasite densities. The original model required the fitting of 38 parameters of malaria epidemiology, either independently (13 parameters) for independent model components, such as within-host parasite dynamics, or through a combined fitting process (25 parameters). A total of 61 scenarios were constructed based on field data and correspond to field sites where the pattern of transmission and one or more epidemiological variables were known. Ten different objective functions (likelihood or sum of squares) were derived from these data sets, representing important epidemiological malaria relationships, such as age pattern of incidence of clinical malaria. A number of alternative model formulations exist.

OpenMalaria was parameterized in accordance with Zambia 2010 Southern Province census data with a simulated population size of N = 10,000, approximately equal to the population of a single health centre catchment. Seasonality was incorporated in the same pattern from Southern Zambian data as in previous publications (e.g. [15]). A warm-up period of one human life span was run to induce a stable level of immunity. During the warm-up period, simulations were run with forced transmission rates. Simulations were run for nominal calendar year t = 2010 until t = 2035 with introduction of RCD at t = 2020 for a period of 10 years. Monitoring was started with surveys in 5-day intervals from t = 2017.

### Model of reactive case detection

**Fig. 1:**
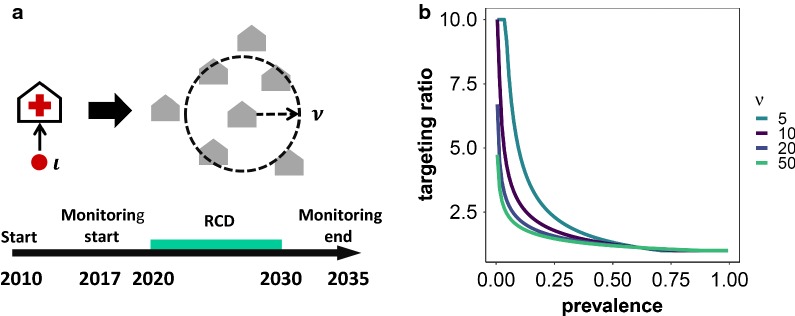
Basic concepts used in the models. **a** Reactive case detection setup. **b** The targeting ratio, τ as a function of prevalence, p and search radius ν

RCD was modelled as a test-and-treat intervention added to and dependent on routine passive case detection and treatment included in the simulations for a period of ten years (Fig. 1a). Within each 5-day time step, for all $\iota$ passively detected index cases tested with a rapid diagnostic test with a detection threshold 50 parasites per 1 μl and a specificity of 0.942 [16], an additional $\nu$ individuals (neighbours) are tested, and treated if infected. All treatments are simulated as leading to an immediate cure (but prophylactic effects were not considered).

OpenMalaria does not explicitly model the spatial pattern of infection. Instead, the effect of case clustering is captured by simulating treatment of all infections in $\nu \tau$ individuals selected at random, where the targeting ratio, $\tau$, is the ratio of the prevalence amongst the $\nu$ individuals closest to the case, to the population prevalence, $p$. Equivalently, $\tau$ is the ratio of the size of a random sample that would be need to be tested (and treated if infected), to the number actually tested and treated, in order to achieve the same number of effective treatments [11].

The targeting ratio was estimated as a function of $p$ and $\nu$ using Markov Chain Monte Carlo (MCMC) methods from data of a cluster-randomized trial carried out in the Zambian study area [11, 17] and is defined as:

$$\tau \left( {\nu ,{\text{p}}} \right) = \exp \left( {\left( { - \alpha_{1} \log \left( p \right) + \frac{{\alpha_{2} }}{\nu } - \frac{{\alpha_{3} }}{\nu }\log \left( p \right)} \right)\frac{N - \nu }{N}} \right) | \tau_{p,\nu } \ge 1$$

where $N$ is the population of the health centre catchment. $\alpha_{1 - 3}$ are constants derived from field data: $\alpha_{1} = 0.230$, $\alpha_{2} = - 1.395$ and $\alpha_{3} = 2.874$. It should be noted that this definition of $\tau$ provides a better fit for low to medium values of $\tau$ and that high values are mathematically possible at low $\nu$ and $p$. Since these have not been observed in the field, $\tau$ was constrained to $\tau \le 10$ (Fig. 1b).

This yields an intervention coverage $c$ of

$$c = \hbox{min} \left( {\frac{{\tau_{p,\nu} \iota \nu }}{N} ,1} \right)$$

where $\iota$ is the actual number of index cases investigated per 5-day period. $\iota$ is dependent on the number of cases that present to a health facility and the maximum capacity of the RCD programme to follow up index cases, $\iota_{max}$. Treatment failure in the community (for whatever reason) or diagnostic insensitivity are equivalent to a reduction in *v.* Coverage was calculated for each 1% prevalence interval and for $\iota = 1 \ldots \iota_{max}$. The number of passively detected index cases that could be followed up per time interval, i.e. the capacity of the program, was constrained by fixing $\iota_{max}$.

### Simulation experiments

*Simulation experiment 1*: a total of 83,200 simulations were run in a full factorial experiment, considering 64 settings defined by different transmission intensities and case management levels for different intensities of RCD with one random seed each (Table 1). The simulated entomological inoculation rates (EIR) were all rather low. Entomological specifications including seasonality were parameterized as in previous simulations of southern Zambia [15]. In accordance with the southern Zambian study site, a reference health care system was chosen such that the probability of effective treatment of any clinical case within 14 days (E_14_) was 21.8% with a failure probability of 19.3% [15]. This was converted to the health care system input parameter in OpenMalaria describing the probability that a case with an uncomplicated episode of malaria seeks care within 5 days as described previously (supplement to Penny et al. [18]). Eight different case management coverages (E_14_ levels) were simulated. The simulated importation rate was zero.

**Table 1:** Setup of simulation experiments

Variable	Description	Levels	Step size	No. of levels in experiment 1	No. of levels in experiment 2
EIR	Entomological Inoculation Rate in infectious bites per person per annum	1, 1.25, 1.5, 1,75, 2.0, 3.0, 4,0, 5.0	–	8	8
E_14_	Probability (%) of effective treatment of any case within 14 days	13.9, *21.8*, 26.0, 36.7, 46.1, 54.6, 62.1 68.9%	–	8	1
$\iota_{max}$	Maximum number of index cases followed up in a 5-day period	1–50	1	50	50
$\nu$	Number of neighbours tested, and treated if infected, for each index case	0–50	2	26	26
Seeds	Seeds for random number sequence	–	–	1	5

The reference value (in italic) for E_14_ is the only value simulated in Experiment 2, where E_14_ is the probability of effective treatment of any clinical case within 14 days

In each of the 64 settings, 1300 RCD strategies were considered (Table 1). The 50 simulations per setting with $\nu$ = 0 were control simulations where no RCD was performed. The simulated total number of infections, number of confirmed clinical cases, and treatments aggregated across all age groups and the simulated EIR, were tracked.

*Simulation experiment 2*: a second simulation experiment was carried out to analyse time to zero prevalence (including both patent infections and those below the detection threshold) as a function of $\iota_{max}$ and $\nu$, replicating each simulation five times with different random seeds (Table 1), allowing us to analyse stochasticity in interruption of transmission. This experiment considered only the reference case management level.

### Analysis of simulation results

Because of the stochasticity featured in the model, the simulated dataset was analysed as though it were a real-world experiment. The initial assessment was of whether and where RCD alone can lead to interruption of transmission as a proof of concept, considering also very low transmission settings (EIR = 1, 1.25). As transmission in such settings can be unstable, stochastic interruption of transmission may occur in control settings, so interruption of transmission in RCD simulations is not necessarily due to the RCD.

*Predictors of interrupting transmission*: The proportion of simulations in Experiment 1 where transmission was interrupted was aggregated across all interventions with EIR as an independent variable, stratified by case management level. The number of simulations summarized per data point, n, was 1300 $\left( {\iota_{max1 - 50 } , \nu_{0 - 50,2} } \right)$. First, the proportion of simulations where RCD interrupted transmission ($p = 0.0$) by the end of the monitoring period (t = 2035) was calculated. Results were aggregated over all runs where RCD was implemented and where the control ($\nu = 0$, i.e. no RCD) did not reach interruption of transmission, regardless of RCD strategy. Thus, the effectiveness of RCD was defined as the proportion $1 - \frac{{e_{1} n_{0} }}{{e_{0} n_{1} }}$, where *e* is the number of scenarios where transmission was interrupted, $n$ is the corresponding total number of scenarios, and the subscripts indicate whether RCD was implemented (subscript 1) or not (subscript 0).

Each of the 64 settings was further assigned to one of three categories depending on whether transmission was interrupted in all, none, or only some simulations. This indicated in which settings success was dependent on the RCD strategy. The proportions or probabilities of interrupting transmission for a given setting and RCD strategy were estimated using a range of classification algorithms (Random Forest, Gaussian Process, Naïve Bayes and Support Vector Machines (SVM)). This provided smooth probability surfaces in the absence of replication, thus avoiding the need for massive numbers of simulations. The performances of these algorithms were compared using 10-fold cross validated mean area under the curve (AUC) and accuracy estimates with a two-third training/test split.

A formal variable importance analysis of the determinants of interruption of transmission was carried out by fitting a set of random forest classifiers to the simulation outcomes for settings where these outcomes were strategy-dependent. Two different random forest classifiers were used based on their inbuilt features, after finding their performance to be very similar (accuracy = 83–84%). The assessments considered the overall permutation importance of EIR, case management, $\iota_{max}$, $\nu$, and the derived variable ${{\iota_{max } } \mathord{\left/ {\vphantom {{\iota_{max } } { \text{max} }}} \right. \kern-0pt} { \text{max} }} \left( {INC} \right)$, the follow up capacity as a proportion of the maximum incidence across the simulation period. The permutation importance of each of the above variables was derived, in each case adjusted for the other variables.

A second approach measuring the overall incremental impact of RCD while adjusting for interruption of transmission in control simulations was to estimate the population attributable fraction, PAF. This is the proportion of simulations with interrupted transmission in Experiment 1 where transmission was interrupted because of the RCD, calculated as described in the supplementary information.

*Median time to zero prevalence in years*: this was computed for each scenario in Experiment 2 where transmission was interrupted in simulations for at least 3 out of 5 random seeds.

*Prediction of RCD success*: the simulations in Experiment 2 were also used to assess the suitability of routinely collected incidence and prevalence data in predicting RCD success (since EIR is not commonly measured in the field). Multiple single-variable logistic regressions was used to determine compare the following variables as predictors of RCD success: pre-intervention EIR, incidence and the prevalence in the year before introduction of RCD, in the first year of the intervention period, and the relative reductions of incidence and prevalence, defined as $\frac{{p_{2020} - p_{2019} }}{{p_{2019} }}$ and $\frac{{INC_{2020} - INC_{2019} }}{{INC_{2019} }}$, where $p_{2019}$, $INC_{2019}$, $p_{2020}$, and $INC_{2020}$ are prevalence and incidence in the last year before and the first year of RCD, respectively.

### Software

The base model variant of OpenMalaria V36 [12] was used. The parameterization process and model variants are detailed by Smith et al. [12, 19]. Scenarios were generated and all analysis was performed in R 3.4.1.The classification analysis was carried out using the mlr package in R. Classif.ranger was used to calculate the overall variable permutation importance, whilst the adjusted permutation importance was calculated using the classif.RandomForest.SCR classifier from the randomForestSCR package (https://kogalur.github.io/randomForestSRC/). Calculations were performed at sciCORE (http://scicore.unibas.ch/) scientific computing core facility at University of Basel.

## Results

Transmission was interrupted in 68.2% of simulations in Experiment 1, 69.3% of simulations with RCD and 42.7% of control settings. The proportion of simulations with interruption of transmission where this could be attributed to the RCD (PAF) was 37.5% overall (95% CI 35.0, 39.9). Results stratified by case management level are presented in Table 2. The effectiveness of RCD ranges between 28% (21, 34) and 51% (41, 59) for different levels of case management. PAF estimates range between 27.4 (20.7, 33.5) at E_14_ = 46.1% and 49.8 (39.7, 58.2) at E_14_ = 13.9%. Overlapping confidence intervals and variability in PAF estimates yield no evidence of a trend for different case management levels. These results demonstrate that RCD can increase chances of interrupting transmission, but also highlight the stochasticity of this.

**Table 2:** Contingency analysis for interruption of transmission with RCD stratified by case management levels

E_14_ (%)	Transmission interrupted		Total		Effectiveness	PAF (%)^a^	p-value**†**
	RCD+ (e_1_)	RCD− (e_0_)	RCD+(n_1_)	RCD− (n_0_)			
13.9	4421	87	10,000	400	0.51 (0.41, 0.59)	49.8 (39.7, 58.2)	< 0.001
21.8 (REF)	5618	130	10,000	400	0.42 (0.33, 0.50)	41.2 (32.4, 48.8)	< 0.001
26.0	6023	121	10,000	400	0.50 (0.42, 0.57)	48.8 (40.7, 55.8)	< 0.001
36.7	6760	150	10,000	400	0.44 (0.37, 0.51)	43.6 (36.1, 50.2)	< 0.001
46.1	7586	218	10,000	400	0.28 (0.21, 0.34)	27.4 (20.7, 33.5)	< 0.001
54.6	7883	224	10,000	400	0.29 (0.22, 0.35)	28.2 (21.8, 34.0)	< 0.001
62.1	8510	191	10,000	400	0.44 (0.38, 0.49)	42.9 (36.9, 48.4)	< 0.001
68.9	8604	244	10,000	400	0.29 (0.23, 0.35)	28.3 (22.6, 33.6)	< 0.001
Total	55,405	1365	80,000	3200	0.38 (0.36, 0.41)	37.5 (35.0, 39.9)	< 0.001

The total number of simulation runs at each case management level is 11,400 with 11,000 RCD+ and 400 RCD−. E_14_ is the probability of effective treatment of any clinical case within 14 days. REF indicates the reference scenario

^a^Wald confidence limits

†Chi-squared

**Fig. 2:**
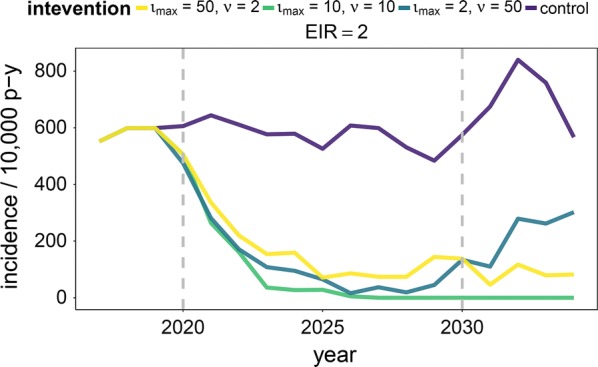
Example of annual incidence throughout the simulation period for different intervention strategies. The total screening capacity of RCD is kept constant at 100, but resources are differentially allocated between maximum number of index cases that can be screened, $\iota_{max}$, and the individuals to be screened within the search radius, $\nu$. Grey dashed lines denote the beginning and end of the intervention period. Case management is at reference level (E_14_ = 21.8%, where E_14_ is the probability of effective treatment of any clinical case within 14 days)

Three example simulations of time series of malaria annual incidence per 10,000 person-years throughout the simulation period are presented in Fig. 2. The overall treatment capacity of the intervention, the product of $\iota_{max}$ and $\nu$, was kept constant at 100 individuals per 5 days but resources were differentially allocated between the two parameters (50 and 2, 10 and 10, or 2 and 50 for $\iota_{max}$ and $\nu$, respectively). The fourth simulation corresponds to treatment of index cases only (zero follow up radius). Only the scenario with equal resource allocation ($\iota_{max} = \nu = 10$) reaches interruption of transmission by the end of the monitoring period, demonstrating the importance of appropriate resource allocation.

**Fig. 3:**
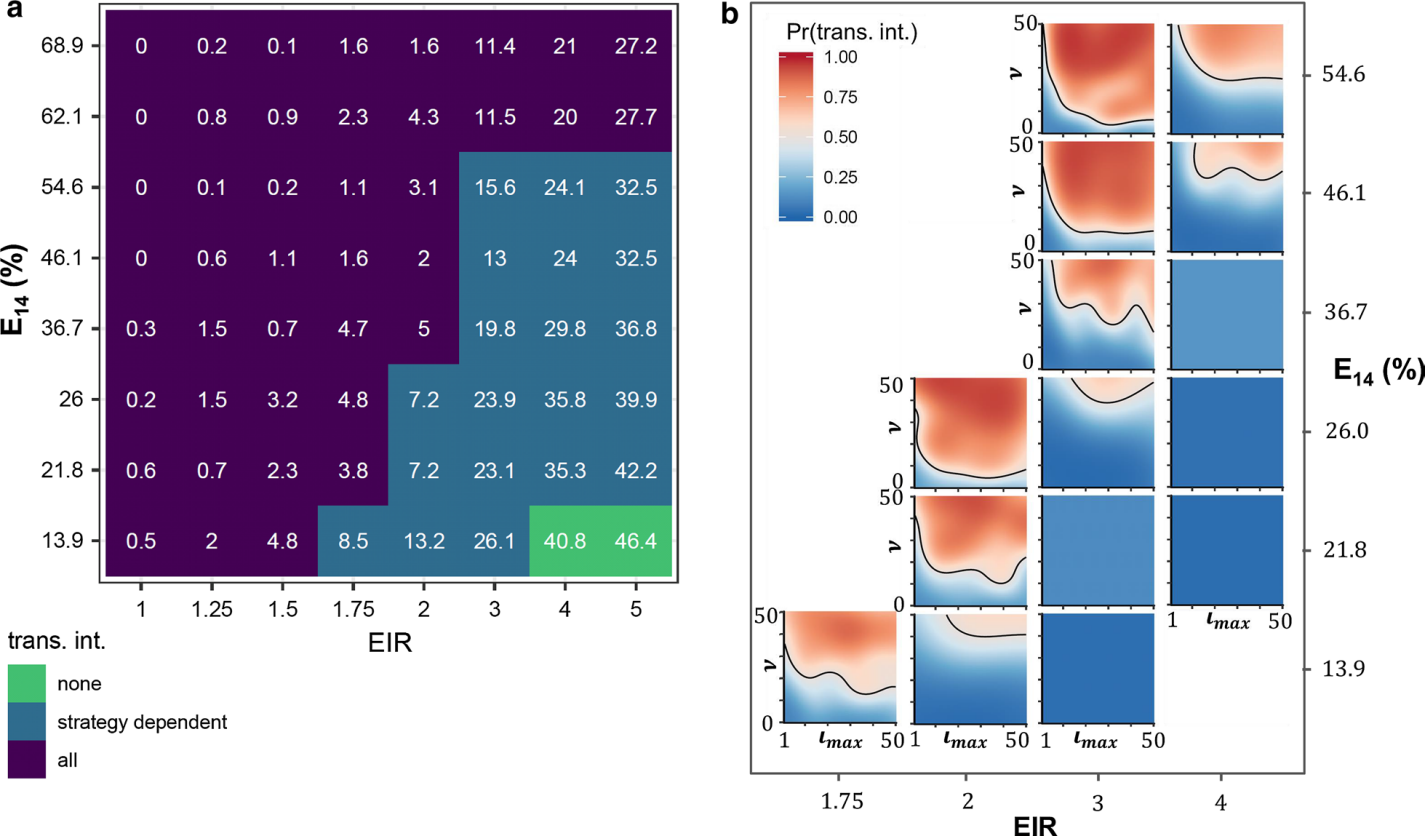
Dependence of prevalence and of probability of elimination on settings. **a** Mean PfPR_0-99_ before RCD for different combinations of transmission potential and case management. Each pixel represents 250 different RCD strategies with the maximum number of index cases that can be followed up, $\iota_{max}$, and the search radius, $\nu$, ranging from 1 to 50 and 0 to 50 respectively. **b** Predictions of probability of elimination for all strategies and settings. Probabilities were generated using a Gaussian Process classifier (c-classification, radial kernel) for each setting where it was previously identified that the elimination outcome is RCD strategy dependent. At EIR = 5 too few simulations reached elimination to generate contour plots. E_14_ is the probability of effective treatment of any clinical case within 14 days

Figure 3a shows settings where transmission was not interrupted in controls categorized by proportion of simulations where transmission is interrupted. Each tile represents simulations of all RCD strategies for the given setting. Categories were assigned based on whether in all, some, or none of the simulations transmission was interrupted. PfPR_0-99_ values indicate mean prevalence prior to RCD.

For all settings where interruption of transmission is dependent on RCD strategy, strategy-dependent probabilities of interrupting transmission were predicted using Random Forest, SVM, Gaussian Process and Naïve Bayes Classifiers. Mean 15-fold cross-validated AUC was highest for Gaussian process classifier (range 0.70–0.98 across settings for 67% training), but predicted patterns were similar across all classifiers. The results are presented in Fig. 3b and demonstrate the narrow range of settings where RCD strategy determines RCD success. The RCD strategy determined the probability of interrupting transmission only in simulations with an initial PfPR_0-99_ of 7–20%, depending on case management levels (for the dependency of initial PfPR_0-99_ on EIR and case management is shown see Fig. S1 in Additional file 1). Below this range, transmission was generally unstable, above this range transmission was too high to be interrupted regardless of RCD intensity. Of the settings where interruption of transmission using RCD was possible, settings with higher transmission or lower management required more intense RCD. High case management alone can lead to 100% interruption of transmission even when transmission is moderate (PfPR_0-99_ = 28%, E_14_ > 63%).

**Fig. 4:**
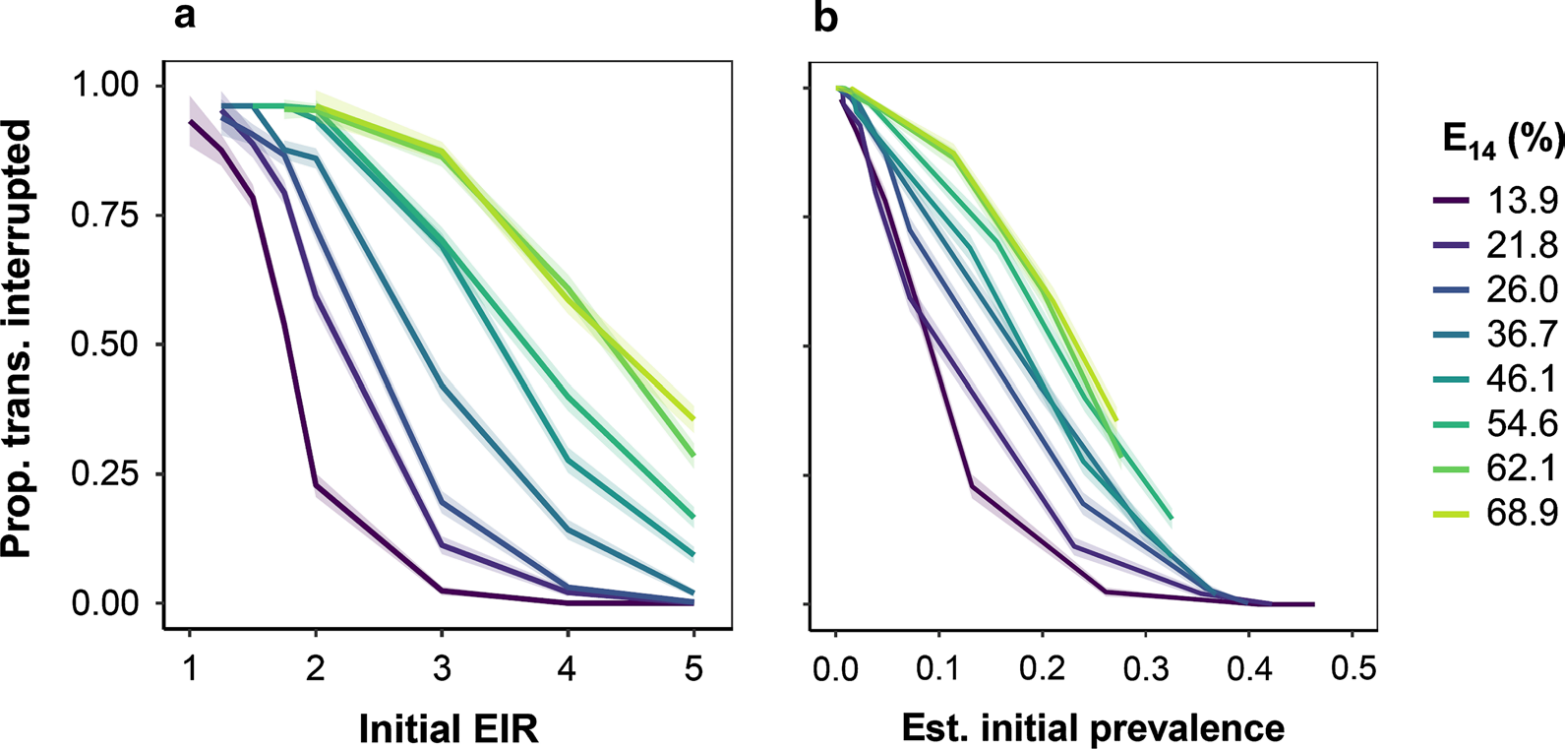
Proportion of runs where transmission is interrupted. The proportion of runs where transmission was interrupted only when RCD was implemented but not in the control setting, where the search radius is 0 ($\nu = 0$). **a** Interruption of transmission by EIR. Proportion of simulations where p = 0 at t = 2035 (points) across all maximum follow-up capacities, $\iota_{max}$ and $\nu$. **b** Interruption of transmission by initial prevalence. Combinations of EIR and case management were converted to mean initial prevalence from simulations. E_14_ is the probability of effective treatment of any clinical case within 14 days

To further assess the differential effects of setting specific parameters, an individual sensitivity analyses for EIR was carried out, stratified by case management level. Figure 4 shows results for different initial EIR, different case management levels, and for the corresponding initial (all age) prevalence. There is a strong inverse relationship between EIR and RCD success. At the reference case management level, 50% of simulations with initial prevalence 10% led to interruption of transmission. Increasing case management can make elimination possible at somewhat higher transmission intensities. RCD with the aim of transmission interruption is thus only appropriate in settings where transmission is very low. Further analyses used machine learning approaches and estimates of the excess probability of transmission interruption that allowed for stochastic interruption of transmission in the absence of RCD.

**Fig. 5:**
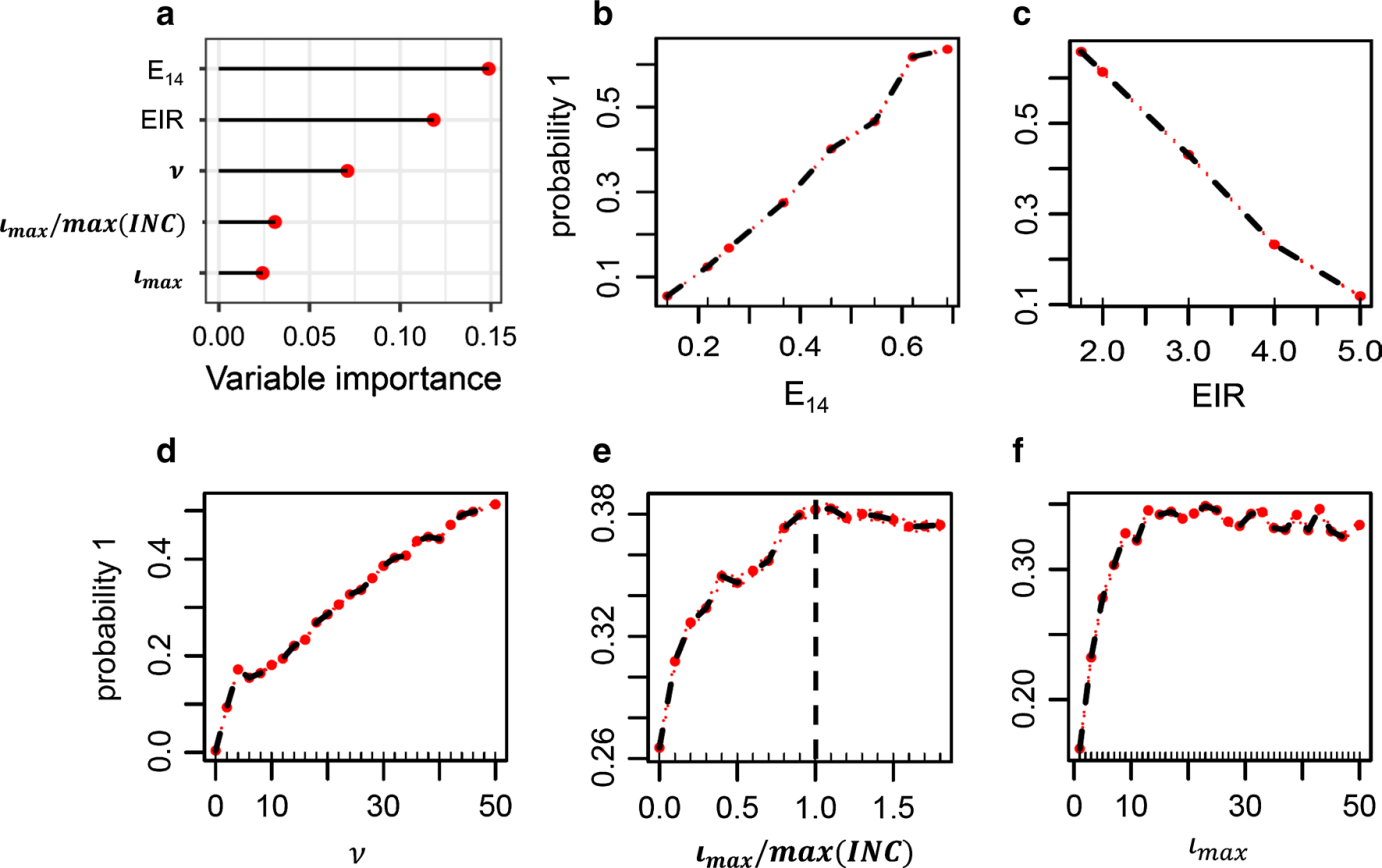
Permutation feature importance in settings with stable transmission (without elimination in controls). **a** Permutation variable importance. **b**–**f** Covariate-adjusted variable importance. All plots were generated using a random forest classifier. “Probability 1” describes the probability of elimination. E_14_ is the probability of effective treatment of any clinical case within 14 days, $\iota_{max}$ and $\nu$ describe the maximum number of index cases that can be followed up and the search radius respectively, and max(INC) describes the maximum 5-day case incidence throughout the simulation period

Figure 5 shows the results of an importance analysis of the effects of $\iota_{max}$ and $\nu$ on the probability of elimination. The results were obtained by fitting a random forest classifier to the simulated data and using an inbuilt function for variable importance. Setting specific parameters, case management and EIR, are the most important variables for elimination (panel A). Panels B-F show the covariate-adjusted variable importance on the probability of elimination. Any increase in search radius is associated with an increase in the proportion of eliminated simulations. Increasing the capacity to treat and follow up index cases ($\iota_{max}$) is of great benefit initially, but quickly reaches an optimum and saturates at a threshold. Increasing case management shifts the saturation threshold to higher values of $\iota_{max}$ but does not change the overall pattern observed with increases of the other parameters.

Further analyses to determine characteristics of the $\iota_{max}$ threshold, were carried out. RCD success in each setting was analysed in relation to the ratio of $\iota_{max}$ to different quantiles of the distribution of 5-day incidence over the entire simulation period from 2020 to 2035. $\iota_{max}$ as a proportion of the all-time maximum incidence, $\frac{{\iota_{max } }}{{{ \hbox{max} }\left( {INC} \right)}}$, showed the strongest relationship with RCD success, suggesting that the ability to cope with maximum incidence during transmission peaks is the most important factor in choosing $\iota_{max}$. It is important to note that the maximum incidence of the simulation period need not be reached prior to RCD implementation as stochastic fluctuations in incidence may lead to an increase in case incidence during the intervention period when case management is low. The adjusted importance analysis of this metric confirms that $\iota_{max}$ should be set such that all index cases can be followed up (Fig. 5f).

**Fig. 6:**
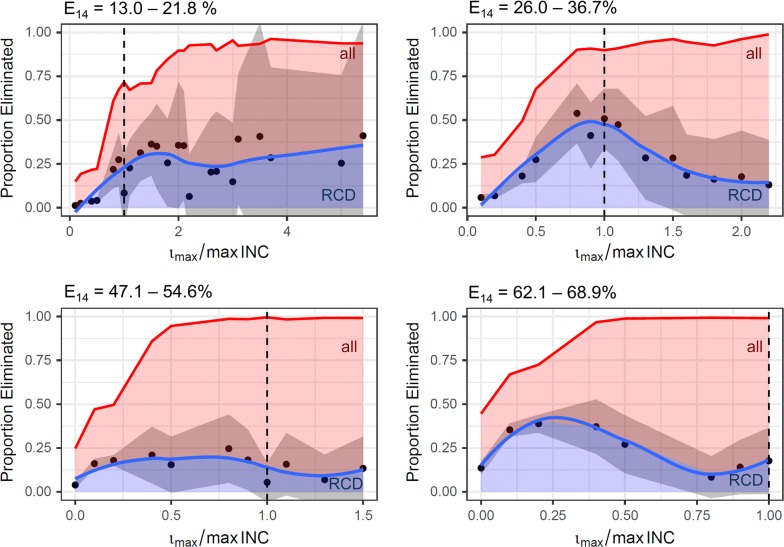
Proportion of eliminated simulations by relative follow up capacity. Blue represents the proportion of eliminated simulations where elimination is attributable to RCD (PAF), relative to the total proportion of eliminated simulations (red). Simulations were aggregated by relative follow up capacity in 10% intervals, thus each data point presents varying numbers of simulations with generally fewer simulations for ratios above 1. Mean PAFs (dots) and 95% CI (gray area) were calculated and a LOESS smoothing function with 0.75 span was fitted through the data (blue line). E_14_ is the probability of effective treatment of any clinical case within 14 days, $\iota_{max}$ is the maximum number of individuals that can be followed up per 5 days, and max(INC) is the maximum 5-day incidence throughout the simulation period

The contributions of case management and case follow up to interrupting transmission were assessed. Figure 6 shows the proportion of simulations where transmission is interrupted ($\frac{e}{n}$) and the proportion where this is attributable to RCD ($PAF\frac{e}{n}$) as a function of $\frac{{\iota_{max } }}{{{ \hbox{max} }\left( {INC} \right)}}$. When stratifying by case management level, the PAF was found to decrease at high case management and high $\frac{{\iota_{max } }}{{{ \hbox{max} }\left( {INC} \right)}}$, although the total proportion of simulations that reach elimination increases. High $\frac{{\iota_{max } }}{{{ \hbox{max} }\left( {INC} \right)}}$ can be a result of both small RCD effort and low transmission. These results thus indicate that at low transmission, the relative contribution of case management to elimination is higher than that of RCD (although RCD may provide some additional benefit).

These results demonstrate that $\iota_{max }$ is optimally chosen such that all index cases can be followed up, even during high transmission seasons. The relative contribution of RCD to interrupting transmission decreases when regular case management alone is strong (at high case management and low transmission). Still, RCD can provide small additional benefit.

**Fig. 7:**
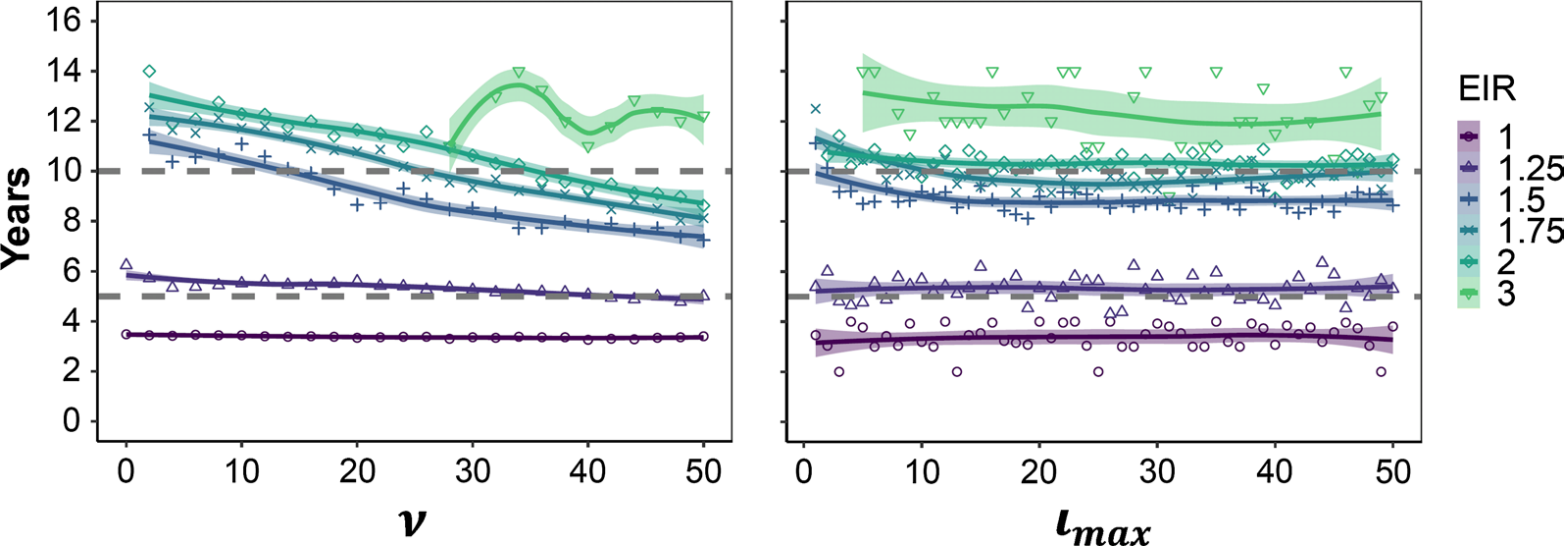
Median time to interruption of transmission. Results are disaggregated by the maximum number of individuals that can be followed up, $\iota_{max}$, and the search radius, $\nu$. The median time to elimination was calculated across all simulations (all values of $\nu$ and $\iota_{max}$, respectively) of the given parameter value. Point estimates were plotted and a LOESS smoothing function was fitted. Median values could not be calculated for EIR > 2 since not enough simulations reached interruption of transmission

Figure 7 summarizes the time to interruption of transmission in years at reference case management level across intervention strategies and transmission levels. Points show the median years to interruption of transmission across five seeds at reference case management level. Presenting the median ensures that > 50% of seeds over all simulations reach interruption and that this is not due to stochasticity. The line and shaded area show the conditional mean and confidence interval of the median, using a LOESS smoother. For EIR = 4 in panel A, confidence intervals were too wide to be displayed. This suggests that transmission is interrupted towards the end of the intervention period, except for at very low transmission. A possible carry over effect beyond cessation of the programme was observed, as at higher EIRs elimination may be reached after the 10-year mark. The intervention strategy makes little difference except for increasing the search radius at EIR 1.5 – 2, approximately equivalent to an initial prevalence of 7%. Increasing $\iota_{max}$ has little effect in determining time to interruption of transmission. Overall, there was a high degree of stochasticity.

**Fig. 8:**
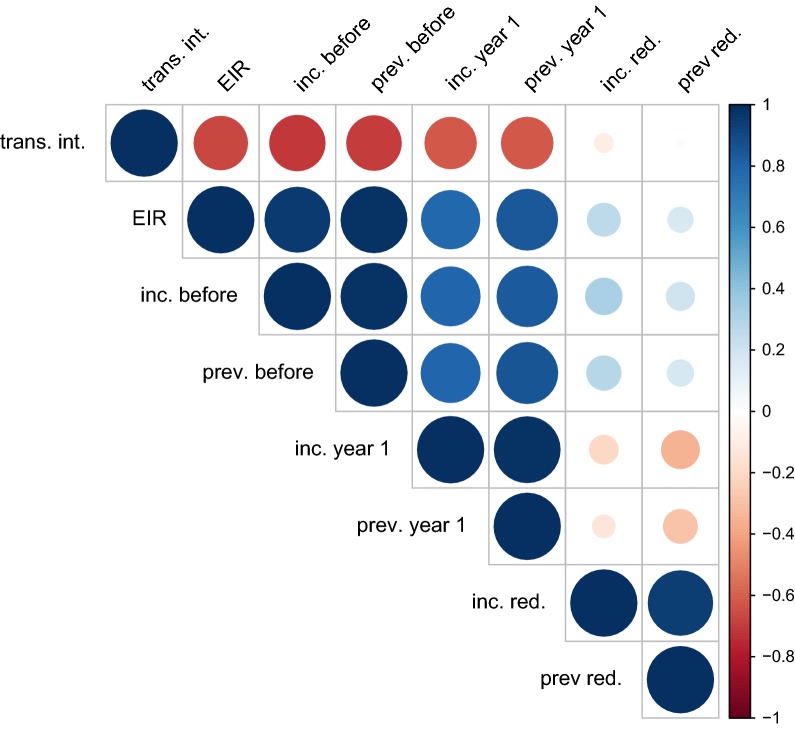
Correlation analysis of predictions of RCD success. Pearson correlation coefficients

Since EIR is difficult to measure in the field, a range of possible predictors of routinely collected data was chosen to assess their predictive ability. The predictive powers of mean incidence and mean annual prevalence the year before onset of the intervention period and in the first year of the intervention period were tested. The predictive ability of the relative reduction in incidence and prevalence in the first year of RCD was also assessed. Prevalence values were log transformed for the purpose of this analysis. The results presented in Fig. 8 suggest that routine data such as incidence and prevalence are equally good in assessing the suitability of a site for RCD as EIR. 83.3% (both) and 86.1% and 84.8% of scenarios were correctly classified using incidence and log prevalence in the year before onset of RCD and the first year of RCD, respectively, as covariates in a single variable logistic regression (Table 3). The cut off points for > 50% probability of interrupting transmission with any strategy at reference case management in the fitted model were 711 per 10,000 person-years, equivalent to a mean of 10 cases per 5 days.

**Table 3:** Single-variable logistic regression results and % correctly classified runs when using the given model

	Estimated odds	95% CI	p-value	Correctly classified (%)
EIR	0.082	(0.079, 0.088)	< 0.0001	83.9
Incidence before	0.996	(0.996, 0.996)	< 0.0001	83.3
log(prevalence before)^a^	0.090	(0.086, 0.094)	< 0.0001	83.3
Incidence year 1	0.990	(0.989, 0.990)	< 0.0001	86.1
log(prevalence year 1)^a^	0.038	(0.035, 0.040)	< 0.0001	84.8
reduction in incidence	0.403	(0.367, 0.443)	< 0.0001	63.4
reduction in prevalence	1.308	(1.164, 1.470)	< 0.0001	62.8

^a^Per 1% increase

## Discussion

A framework for implementing spatially targeted reactive interventions in OpenMalaria was developed by approximating a targeting ratio which captures spatial heterogeneity. Reactive case detection only leads to local interruption of malaria transmission in settings with low transmission potential, measured by either EIR or prevalence (initial EIR approx. 1-3 or prevalence approximately < 7–19% for 50% probability of success, depending on case management levels). RCD was effective in 38% (95% CI 36, 41) of scenarios simulated, with a small trend towards greater effectiveness at lower case management. Large and overlapping confidence intervals highlight the stochasticity of interrupting transmission. Whilst the simulations use transmission intensity in terms of EIR and 5-day case management levels as inputs, they show that qualifying settings are also identifiable using routinely collected prevalence and incidence data. It follows that settings can be classified into suitable or non-suitable for RCD, and predictions can be made such that RCD is not initiated where it is very unlikely to be successful. This is roughly consistent with another modelling study based on the same Zambian data, which suggests a prevalence threshold for RCD of approximately 10% [20]. The relative success of RCD (in terms of reduction in incidence or prevalence) in the first year of implementation is not a good predictor of ultimate success.

A primary objective of this study was to determine which operational characteristics of RCD most influence the effectiveness. Considering the joint and individual influences of increasing capacity to follow up index cases and the search radius, a larger search radius is always beneficial. In contrast, increasing $\iota_{max}$ only increases the probability of success up to a certain threshold, $\iota_{max}^{*}$, with no additional value in increasing it beyond this. This threshold is related to the maximum number of cases presenting to be treated, and defines a cut-off point in case incidence beyond which the setting is not suitable for elimination via RCD altogether. For settings with low to intermediate case management levels, all index cases should be followed up. A natural approach for programme managers to use when considering introducing RCD, is to initiate it when the number of cases reporting has fallen to the level where the capacity exists to follow them all up. The simulations endorse this instinct, in contrast to previous modelling of RCD that used a deterministic compartmental susceptible-infected-susceptible (SIS) model with the same function for the targeting ratio [11]. The SIS-models attributed greater importance to following up more index cases, even at the cost of a smaller $\nu$ across the entire parameter space, as the targeting ratio is much higher at small $\nu$. In that analysis the upper bound of the possible number of index cases was set by the standing crop of infections; it was assumed that any pre-defined number of index cases less than this can be found.

This paper takes a slightly different programme planning focused approach by assessing the importance of the follow-up capacity rather than the actual number of followed up index cases. Cases that do not seek official care or are asymptomatic limit the potential number of index cases. When the case management level is increased, more index cases become available making it useful to adjust $\iota_{max}$ upwards. Thus it would seem that case management limits the settings where elimination is possible because it imposes the threshold effect on $\iota_{max}$.

Despite providing a proof of concept that RCD can lead to local elimination, the results also suggest that successful RCD is highly resource intense and likely to be very costly (though no formal economic evaluation is presented). In most suitable settings RCD would have to be conducted for more than 5 years with a relatively aggressive strategy to yield a probability of interruption of transmission of > 50%. In the area of the trial in Zambia, for example, the weighted mean EIR is estimated to be approximately 2.9 [18] and the case management (*E*_14_) at the time where the RCD trial was conducted was about 21.8% [15]. In such a setting, elimination with RCD is not feasible. Today, Zambia’s case management (E_14_) is estimated to be 34.7%. Such a setting would require a follow-up capacity of approximately 10 index cases and search radius of > 35-40 individuals, i.e. a total screening capacity of 350-400 individuals per 5-day period would be necessary in order to reach interruption of transmission within 10 years with a probability of success of only 50%. On the contrary, if case management was further increased to E_14_ = 46% [21], following up 5 index cases with a 25-person radius, i.e. 125 individuals in total, would suffice for a 50% probability of interrupting transmission. If case management was to be increased to 62% this alone would lead to interruption of transmission (in all simulations). Strengthening access to care would also have further positive implications on population health and likely lower the burden of disease not just of malaria, but overall. Previous modelling studies having assessed RCD come to similar conclusions. An independent modelling study [22] suggests that in low prevalence settings, improving case management may be more impactful than RCD, although RCD may bring qualitative benefits to a setting. For example, RCD may improve visibility of community health workers and raise awareness of the disease in general.

The results indicate that policy decisions should prioritize improving access to care followed by appropriate treatment and follow up of all index cases. This can be explained through a greater importance of following up as many as possible *definite cases* that present to the health facility. All index cases are definitely cases (*definite cases*) whereas individuals in the search radius may or may not be cases at probability $p \tau \left( {p,\nu } \right)$. Targeting is stronger and $\tau$ is larger at small $\nu$. The per-person probability of being a case in the radius around an index case is thus greater the smaller the radius. Increasing the number of index cases through improving access to care and treating and following up these index cases is therefore always more targeted than treating individuals in the search radius. RCD is thus only worthwhile where the number of cases detected through RCD $\left( {\hbox{min} \left( {\iota , \iota_{max} } \right) \nu p \tau } \right)$ and in the limit, as $\iota_{max} \to \iota$, the number of cases detected per index case is greater than 1 $\left( {1 < \nu p \tau \left( {p,\nu } \right)} \right)$. This condition describes the threshold at which more hidden cases are found through RCD than index cases present to the health facility. If the aim is local elimination, the second condition for implementing RCD must of course be that interrupting transmission in the setting is itself feasible. As it is a highly stochastic event, this condition is more difficult to predict. Together, these two conditions define the narrow range of settings in which interrupting transmission through RCD alone is feasible. Where it is possible, it will thus generally be more effective to increase case management rather than implementing RCD. Based on these results, the following prioritization is proposed: case management to increase number of index cases ≫ following up all index cases ≫ increasing the search radius. The effect of increasing the search radius also likely flattens off at some point, but not within the parameter space considered.

The results are based on Zambian parameterization and thus the transferability of results across settings with different population densities remains to be confirmed. However, by defining the search radius in terms of number of individuals rather than a physical distance, the findings presented here should be more translatable across settings at different population densities, assuming that vector movement and thus disease spread is dependent on host availability more so than physical distance. Future research comparing RCD in different settings should explore this hypothesis. The study did not consider case importation. Simulations of Zambian settings with low coverage of case management [22] conclude that this is an important determinant of whether elimination is achieved, but simulations with OpenMalaria suggest that this is not the case if case management coverage is high (Smith et al. pers.commun.). Whilst OpenMalaria does consider heterogeneous populations, including in their health-seeking, the reactive intervention is applied to random individuals in the population.

The targeting ratio approach presented here provides a framework for implementing generic spatially targeted reactive interventions in OpenMalaria. Future work should adapt the framework to different settings by fitting the targeting ratio to data from different settings. However, as long as infections are acquired within the community similar patterns to those presented here can be anticipated, since the search radius is specified in terms of number of individuals, thereby making it independent of the population density. Future investigations may further include incorporating importation of cases as well as varying the timing of RCD and implementing RCD in combination with other (mass) interventions. If RCD is only successful at low transmission it would be well worth investigating adding a mass intervention at the beginning of RCD intervention course as well as employing RCD in scenarios where mass interventions have brought EIR down to < 3 infectious bites per person per year. Further, one may investigate employing RCD seasonally in the dry season when incidence is low. This may lead to stochastic elimination and save resources. This framework can also be adapted and used for wide ranges of spatially targeting interventions, such as RCD in different settings and reactive vector control.

## Conclusions

Overall, the study demonstrates that RCD can increase the chances of stochastic elimination, but that it is a very resource intense, such that other interventions are likely more appropriate in most settings. In its final stages, RCD leads to a sustained reduction in overall burden of malaria through strengthening the health care system so that imported infections are controlled. This stabilizes the disease-free state [11], in contrast to *one*-*off* higher impact interventions, such as MDA that have strong immediate effects over a limited period but do not provide a sustained reduction in transmission. With MDA, even in the most favourable circumstances, persistence is highly stochastic depending on the size of the residual reservoir of infectious people exposed to mosquitoes. However, despite its potential impact RCD is a highly resource intense, long-term intervention that is inappropriate in many settings where resources are limited. In such settings, investments may be better made in improving the routine health care system.

## Additional file

**Additional file 1.** Additional figure.

## Data Availability

The data and underlying code are available on request from the authors.

## Abbreviations

EIR: entomological inoculation rate
MDA: mass drug administration
RCD: reactive case detection
PAF: population attributable fraction
E_14_: probability of effective treatment of any clinical case within 14 days
