# Supplementary figures and images for: Modelling reactive case detection strategies for interrupting transmission of Plasmodium falciparum malaria
Source: Malar J. 2019 Jul 30;18:259. doi: 10.1186/s12936-019-2893-9 (PMC6668148; doi:10.1186/s12936-019-2893-9)

Case Management (%)

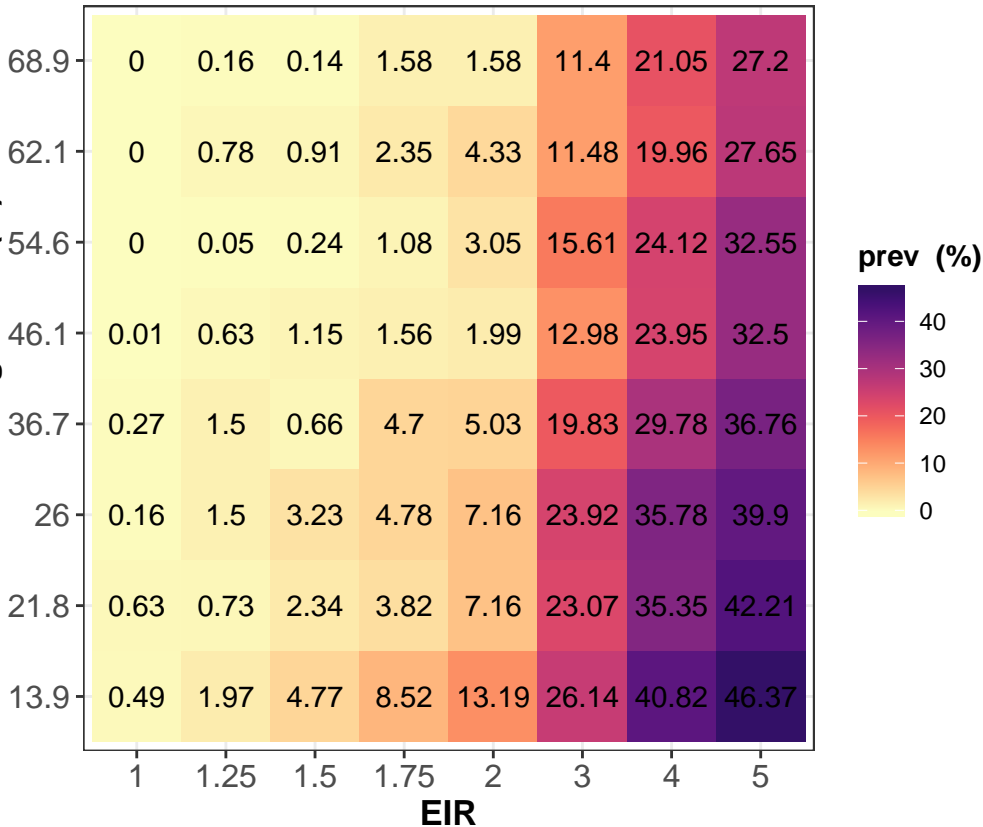

Supplement: Supplementary file 1 — Additional file 1. Additional figure. [file 12936_2019_2893_MOESM1_ESM.pdf]
